# Effect of low-dose hydrocortisone on the expression of glucocorticoid receptor alpha of the septic kidney in rats and its protective effect on kidney injury

**DOI:** 10.1186/cc10763

**Published:** 2012-03-20

**Authors:** DW Wu, HP Guo

**Affiliations:** 1 Qilu Hospital of Shandong University, Jinan, Shandong, China

## Introduction

Inflammation out of control caused by sepsis can eventually lead to multiple organ dysfunction, of which the kidney is one of the most common injured organs. Sepsis-induced acute kidney injury (SI-AKI) can obviously increase the mortality of sepsis. At present, there are controversial views about the impact of exogenous glucocorticoid to SI-AKI on kidney pathological changes and glucocorticoid receptor (GR) expression. So, we want to investigate whether low-dose glucocorticoid has a protective effect on SI-AKI and what is the mechanism.

## Methods

Healthy Wistar male rats were randomly divided into a sham group, SI-AKI group and SI-AKI hydrocortisone group (HC group). The SI-AKI model was reproduced using the cecum ligation and puncture method. Pathological changes of the kidney were detected by H & E staining. The expression of GRα and NF-κB in the kidney was detected by immunohistochemistry. The levels of IL-1, IL-6, TNFα and IL-10 in the plasma were detected by ELISA.

## Results

The survival rate of the AKI group and HC group showed no statistical difference (*P *>0.05). H & E stain showed renal tubular epithelial cells swelling and falling off, and the tubular brush border disappeared and vacuolated in the AKI group. Pathological changes of the renal tubular could be alleviated after hydrocortisone treatment. Compared with the AKI group, immunohistochemistry showed that GRα expression was increased and NF-κB expression was decreased in the HC group (*P *< 0.01). The level of TNFα, IL-6, and IL-1 were reduced and the level of IL-10 was increased in the HC group compared with the AKI group (*P *< 0.01).

## Conclusion

Low-dose hydrocortisone can inhibit the NF-κB activity, possibly in part by increasing the expression of GRα in renal sepsis rats. Accordingly, it could reduce the production of inflammatory factors participating in sepsis, effectively inhibit the inflammation and extenuate the sepsis-induced renal pathological changes.
